# Machine learning-based identification of diagnostic and prognostic mitotic cell cycle genes in hepatocellular carcinoma

**DOI:** 10.1371/journal.pone.0331118

**Published:** 2025-08-28

**Authors:** Ceren Sucularli

**Affiliations:** Department of Bioinformatics, Institute of Health Sciences, Hacettepe University, Ankara, Turkey; Indiana University School of Medicine, UNITED STATES OF AMERICA

## Abstract

Mitotic cell cycle (MCC) is a critical process in cell growth and division, and dysregulation of MCC genes may contribute to tumorigenesis. In this study, to identify diagnostic and prognostic value of MCC genes, differentially expressed MCC genes between HCC and normal tissues were identified and subjected to machine learning methods. SVM-RFE and RF-RFE were employed to select the most informative diagnostic genes. The SVM-RFE model demonstrated high performance in TCGA (AUC = 1.0), and generalizability across GSE77509 (AUC = 0.95) and GSE144269 (AUC = 0.879), outperforming RF-RFE. Permutation testing confirmed that these AUCs were outside the null distribution for all datasets. Nine genes, CDKN3, TRIP13, RACGAP1, FBXO43, EZH2, SPDL1, E2F1, TUBE1 and CDC6, were common in SVM-RFE and RF-RFE and showed robust individual diagnostic performance across datasets (AUCs > 0.81). Univariate Cox regression followed by LASSO Cox regression was used for identification of prognostic gene signature consisted of eight MCC genes, BCAT1, DPF1, CDKN2B, CDKN2C, TUBA3C, IGF1, CDC14B and SMARCA2, that predicted overall survival of HCC patients. The risk score was shown to be an independent prognostic factor for HCC and its combination with AJCC stage improved prognostic value. Kaplan–Meier analysis showed that high-risk score was associated to poorer survival across clinical subgroups; stage, grade, age, and gender. Additionally, risk score was significantly higher in patients with advanced-stage and high-grade tumors. In conclusion, diagnostic biomarker candidates classifying HCC patients and healthy controls, and a novel prognostic gene signature predicting overall survival of HCC patients were identified by using machine learning approaches.

## Introduction

Mitotic cell cycle (MCC) is a precisely regulated process, which includes DNA replication, chromosome segregation and cell division [1]. Because of its importance, this process is tightly monitored by surveillance mechanisms, which ensures the accuracy and correct order of the cell cycle. Defects in proper cell cycle progression leads to deregulated cell proliferation, genomic instability and eventually tumorigenesis [2]. Gene expression signatures constituted with genes functioning in cell cycle and DNA damage response have offered promising prognostic markers for HCC [3,4]. The significance and clinical utility of gene expression profile-based assays, such as MammaPrint and Oncotype DX, have been established in breast cancer for predicting cancer outcome and guiding decision making in therapy [5–7]. This highlights the potential value of identifying diagnostic and prognostic genes for other cancers, such as HCC, to assist clinical practice.

Liver cancer is the 3^rd^ leading cancer in deaths caused by cancer and 6^th^ in number of new cases [8]. The most common type of liver cancer is hepatocellular carcinoma (HCC) [9]. Different risk factors and mechanisms have been associated to HCC, including activation of oncogenic signaling pathways or altered functions of cancer driver genes due to mutations [9]. In addition to genetic variations, gene expression alterations have been identified as an important factor in HCC pathogenesis.

Machine learning (ML) algorithms, including support vector machine recursive feature elimination (SVM-RFE) and random forest with recursive feature elimination (RF-RFE), have been used to predict potential diagnostic cancer biomarkers by using existing transcriptome data [10–12]. SVM-RFE has been shown to be a powerful algorithm for identifying biologically relevant genes during feature selection [13]. RF is another method that has been used for feature selection in cancer diagnosis [12]. Combining RF with RFE improves feature selection, due to the addition of RFE, which iteratively removes least important features to yield more relevant features [14]. Least absolute shrinkage and selection operator (LASSO) is a variable selection and shrinkage method, which has been used to predict the survival of cancer patients and establishment of prognostic gene signatures [3,15,16].

Due to the significance of MCC in cancer progression, targeting cell cycle genes, which are associated with tumorigenesis and patient outcome, may facilitate detection of cancer formation, create therapy strategies to improve patient prognosis and provide new targets for therapeutic applications. Therefore, in this study MCC genes that were differentially expressed in HCC were used the identify diagnostic biomarker candidates and construct the prognostic MCC gene signature.

## Materials and methods

### Data retrieval and analysis

RNA-seq count data of The Cancer Genome Atlas Liver Hepatocellular Carcinoma (TCGA LIHC) tumor and normal samples (primary solid tumor, n = 371, and solid tissue normal, n = 50) and matching clinical data were downloaded by using *TCGAbiolinks* R/Bioconductor package [17]. RNA-seq count data of tumor and normal samples from GSE77509 (tumor, n = 20, and normal, n = 20) [18] and GSE144269 (tumor, n = 70, and normal, n = 70) [19] were downloaded from GEO database (https://www.ncbi.nlm.nih.gov/geo/, [20]). Genes with low counts were filtered and raw counts were TMM normalized by using *edgeR* [21] and *limma* [22] R/Bioconductor packages. GSE14520 [23] raw data from tumor tissue of HCC patients (n = 221) and related clinical data, downloaded from GEO database [20], were preprocessed and RMA normalized by using *oligo* R/Bioconductor package [24]. MCC gene set; GOBP_MITOTIC_CELL_CYCLE, and the transcript names in this gene set were retrieved from the Molecular Signatures Database (MSigDB v2023.2.Hs, https://www.gsea-msigdb.org/gsea/msigdb/index.jsp, [25,26]).

### Identification of differentially expressed MCC genes in TCGA LIHC and GSE77509

Differentially expressed genes (DEGs) between tumor and normal samples of TCGA LIHC and GSE77509 were identified. Genes with adjusted p-value < 0.05 and |log fold change| > 1 were considered as significant in each dataset. To obtain the differentially expressed MCC genes, the upregulated and downregulated DEGs from both datasets, and MCC genes from GOBP_MITOTIC_CELL_CYCLE gene set were compared via venn diagram and 183 intersection genes were selected for further analysis (S1 Fig).

### Feature selection using SVM-RFE and RF-RFE

To identify most informative MCC genes for HCC classification, the intersection genes were subjected to ML algorithms, SVM-RFE and RF-RFE. TCGA LIHC was used as training dataset, GSE77509 as internal validation set and GSE144269 as external validation set. Prior to application of ML algorithms, a filtering step was applied on TCGA LIHC to remove highly correlated genes (Pearson r > 0.9). To increase the robustness of the feature selection process, RFE with 10-fold cross validation for 50 iterations was performed. Genes that were selected in at least 90% iterations were subjected to a final round of feature selection using RFE with 10-fold repeated cross validation with 5 repeats to obtain most relevant diagnostic genes for training of SVM with linear kernel and RF models. To evaluate generalizability of models and prevent overfitting, a nested cross-validation and permutation testing (n = 100) were performed. Model performance was evaluated with AUC, sensitivity, specificity, accuracy, precision and recall. Analyses were performed by using *e1071*, *caret* [27], *ROCR* [28], *pROC* [29] and *randomForest* [30] R/Bioconductor packages.

### Construction and performance of the prognostic MCC gene signature with LASSO

The intersection genes were utilized to construct a prognostic gene signature. TCGA LIHC patients with follow up time ≥ 30 days and complete information of vital status and follow up time were included in survival analysis and construction of gene signature. GSE14520 cohort was used to validate the prognostic MCC gene signature. Quantile normalization was applied on TCGA dataset and harmonization across datasets was performed as previously described [31,32]. Univariate Cox regression was conducted to evaluate the association between MCC genes and overall survival in TCGA LIHC. Clinical information (including days to death, days to last follow up and vital status) was used to calculate survival time and censoring status. For each gene, Hazard ratios (HR), log hazard ratios (logHR), standards errors, 95% confidence intervals (CI) and p-values were calculated. The proportional hazards (PH) assumption was tested by using Schoenfeld residuals and genes violating the PH assumption (p ≤ 0.05) were excluded from further analysis. To control multiple testing, p-values were adjusted by using Benjamini-Hochberg FDR method. Genes did not violate the PH assumption (PH p > 0.05) and with FDR < 0.05 were retained for further analysis. To further validate the reliability of selected genes, pairwise Pearson correlations among the significant genes were examined and gene pairs with correlation coefficients > 0.9 were considered as highly correlated.

The significant genes from univariate Cox regression analysis (FDR < 0.05 and PH p > 0.05) were used as input for LASSO Cox regression analysis with 10-fold cross-validation. Genes with nonzero LASSO coefficients were used construct the prognostic model. A risk score was calculated for each patient as follows:


$$\text{ Risk Score}=\sum_{i=1}^{n}(\text{coef}i\;\times \text{ Exp}i)\;\text{\textbackslash{} \textbackslash{} \textbackslash{} \textbackslash{} \textbackslash{} \textbackslash{} \textbackslash{} \textbackslash{} \textbackslash{} \textbackslash{} \textbackslash{} \textbackslash{} \textbackslash{} \textbackslash{} \textbackslash{} \textbackslash{} \textbackslash{} \textbackslash{} \textbackslash{} \textbackslash{} \textbackslash{} \textbackslash{} \textbackslash{} \textbackslash{} \textbackslash{} }$$ (1)

Where n is the number of genes in the prognostic model, coef is coefficient for the gene*i*, and Exp is gene expression level of gene*i*. Accordingly, patients were divided into low- and high-risk groups based on the median value of risk score. In addition, the optimal cutoff to divide patients into low- and high-risk groups was calculated by maximally selected rank statistics. Kaplan-Meier survival analysis was performed to compare the overall survival rate between the low- and high-risk groups for median and optimal cutoff. The prognostic model was assessed by receiver-operating characteristic (ROC) curves and AUC values for 1-, 3- and 5-year survival were reported. ROC curves at 1-, 3-, and 5-year time points were generated using the timeROC package, which applies the marginal weighting method to account for right-censored survival data when estimating AUC values.

Univariate and multivariate Cox regression analyses were executed to evaluate the independent predictive value of the signature from clinical parameters, including American Joint Committee on Cancer (AJCC) pathologic stage, grade, age and gender. For each clinical parameter and risk score, HRs, 95% CIs, and p-values were computed and to control for multiple hypothesis testing, FDR adjustment was applied using the Benjamini-Hochberg method. Additionally, the PH assumption was tested for each covariate using Schoenfeld residuals for univariate and multivariate Cox regression. The concordance index (C-index) was calculated for three models: stage alone, risk score alone, and a combined model with both stage and the risk score. The prognostic value of the gene signature and the distribution of the risk score were assessed in subgroups stratified by clinical parameters, including AJCC pathologic stage, grade, age and gender. Analyses were performed by using *survival* [33], *survminer, glmnet* [34], *timeROC* [35] and *survivalROC* [36] R/Bioconductor packages.

## Results

### Identification of the diagnostic biomarker candidates from differentially expressed MCC genes by using SVM-RFE

GOBP_MITOTIC_CELL_CYCLE gene set was consisted of 932 unique MCC genes. Among the 932 MCC genes, 183 genes, 147 upregulated and 36 downregulated, were identified as differentially expressed in both TCGA LIHC and GSE77509 (adj. p-value < 0.05 and |lfc| > 1).

**Fig 1:**
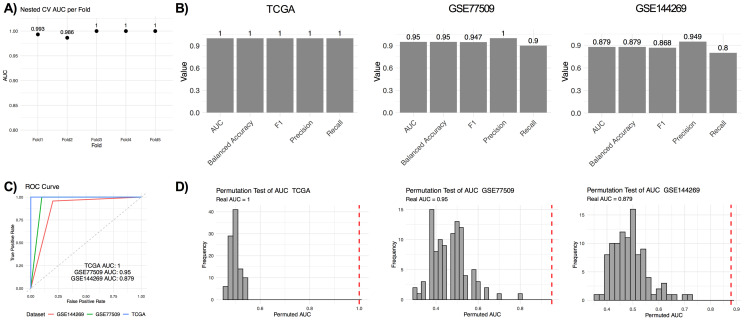
The performance of SVM-RFE. A) Nested 5-fold cross-validation AUC values for the TCGA LIHC. B) The performance of SVM-RFE based on the confusion matrix metrics in TCGA LIHC, GSE77509 and GSE144269. C) ROC curve of three datasets. D) Permutation tests (n = 100) of AUCs for TCGA LIHC, GSE77509 and GSE144269. Real AUCs were shown as red dashed lines.

To identify the most informative MCC genes for distinguishing tumor from normal samples, SVM-RFE using a linear kernel was applied. After filtering highly correlated genes, performing RFE with 50 iterations, and a final RFE step using SVM, 110 genes were selected for model training. The model achieved an AUC of 1.0 in the training set, TCGA LIHC, and a nested 5-fold cross-validation confirmed the model’s robustness (mean AUC = 0.996, SD = 0.006; Fig 1A). The model showed consistent high accuracy, F1-score, precision and recall across the TCGA, GSE77509 (internal validation set) and GSE144269 (external validation set) (Fig 1B). The AUC values of TCGA LIHC, GSE77509 and GSE144269, 1, 0.95 and 0.879, respectively, indicated a generalizability across datasets (Fig 1C). Permutation tests (n = 100) further validated the model by demonstrating that observed AUCs lay far outside the null distribution for all three datasets and none of the permuted AUCs reached the observed AUCs (permutation test empirical p value <0.01, Fig 1D).

### Identification of the diagnostic biomarker candidates from differentially expressed MCC genes by using RF-RFE

To evaluate the diagnostic value of differentially expressed MCC genes in HCC, another classifier, RF, was employed with RFE. Filtering highly correlated genes, followed by RFE using RF with 50 iterations, resulted in 28 genes. These genes were then subjected to final RFE to obtain 15 genes.

**Fig 2:**
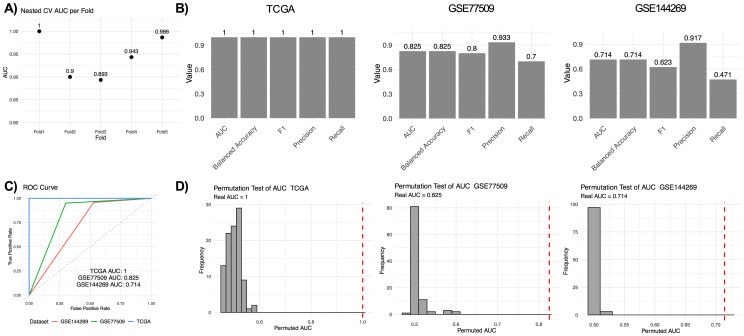
The performance of RF-RFE. A) Nested 5-fold cross-validation AUC values for the TCGA LIHC. B) Confusion matrix metrics for TCGA LIHC, GSE77509, and GSE144269. C) ROC curve for TCGA LIHC, GSE77509, and GSE144269. D) Permutation testing (n = 100) results of all three datasets. Red dashed lines represented real AUC values.

Nested 5-fold cross-validation for TCGA confirmed model consistency with a mean AUC and SD of 0.945 ± 0.049 (Fig 2A). Model performance declined in the GSE77509 and GSE144269 datasets relative to TCGA, as reflected by balanced accuracy, recall and AUC values (Fig 2B and Fig 2C). Similar to SVM-RFE, the discriminative ability of the model was confirmed with permutation testing (n = 100) (permutation test empirical p value <0.01, Fig 2D).

### Selection of the most relevant features for diagnosis of HCC

**Fig 3:**
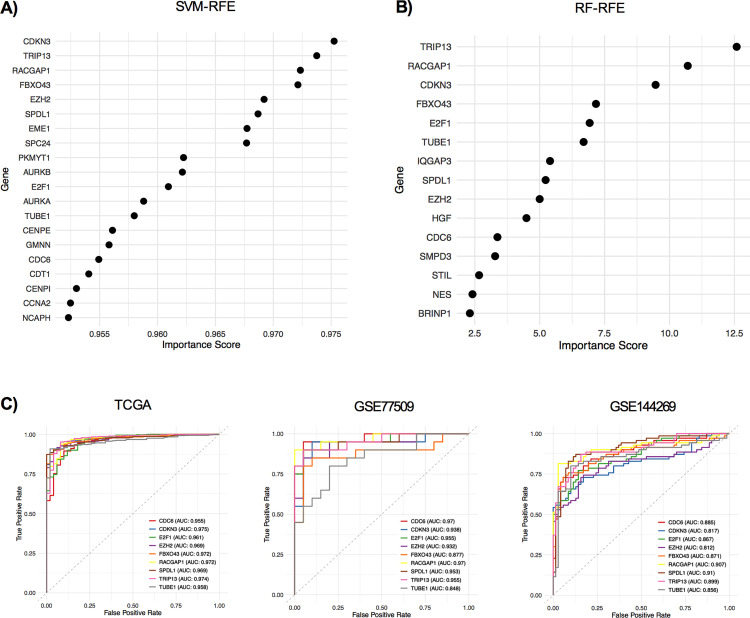
Important features from SVM-RFE and RF-RFE. A) Top 20 features of SVM-RFE. B) 15 features of RF-RFE. C) ROC curves showing the diagnostic performance of the nine shared genes.

To select the most relevant and important features for diagnosis of HCC, top 20 features of SVM-RFE and 15 features of RF-RFE were investigated. Although the ranking was varied, nine genes; cyclin dependent kinase inhibitor 3 (CDKN3), thyroid hormone receptor interactor 13 (TRIP13), Rac GTPase activating protein 1 (RACGAP1), F-box protein 43 (FBXO43), enhancer of zeste 2 polycomb repressive complex 2 subunit (EZH2), spindle apparatus coiled-coil protein 1 (SPDL1), E2F transcription factor 1 (E2F1), tubulin epsilon 1 (TUBE1) and cell division cycle 6 (CDC6), were common between classifiers (Fig 3A and Fig 3B).

In TCGA, all nine genes achieved AUCs > 0.95, indicating excellent diagnostic separation between tumor and normal samples. All genes maintained good to excellent performance with AUCs > 0.81 in GSE77509 and GSE144269. These results supported the robustness and potential value of these genes as individual diagnostic biomarker candidates for HCC (Fig 3C).

### Construction of prognostic MCC gene signature with LASSO

**Fig 4:**
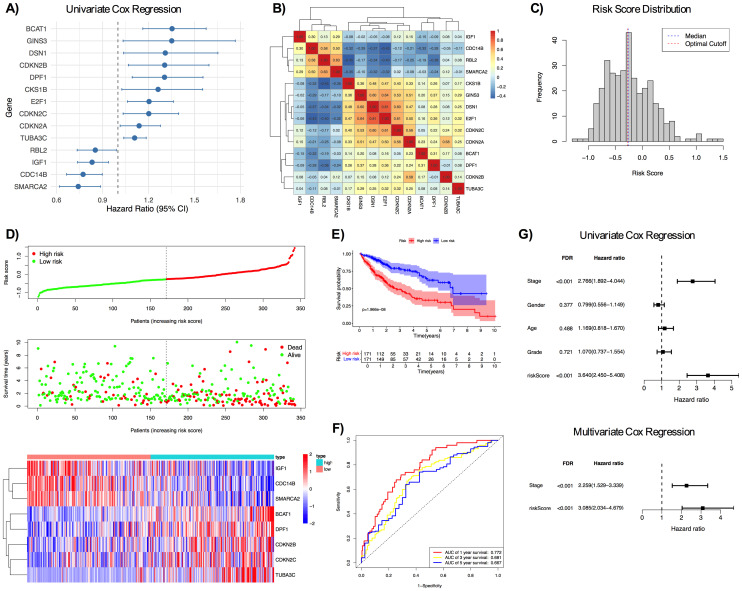
Evaluation of the prognostic performance of MCC gene signature in TCGA LIHC. A) Forest plot for 14 genes significantly associated with overall survival in TCGA LIHC (FDR < 0.05, PH p > 0.05) B) Pairwise Pearson correlation heatmap of the 14 significant genes. C) Distribution of risk score, blue dashed line and red dashed line represented median and optimal cutoff, respectively. D) Distribution and survival information of the risk score and heatmap of the gene expression of 8 genes. E) Kaplan-Meier analysis of TCGA LIHC patients. F) ROC curve of TCGA LIHC patients. G) Univariate Cox regression for stage, age, gender, grade, and risk score and multivariate Cox regression for stage and risk score.

Univariate Cox regression was performed with differentially expressed MCC genes that were represented in GSE14520 dataset to identify the MCC genes associated with overall survival of HCC patients. Accordingly, 14 genes were found to be significantly associated with overall survival based on FDR < 0.05 and PH p > 0.05, ensuring that all genes satisfied the PH assumption. Based on the HR values, four genes with HR < 1 were detected as protective and ten genes with HR > 1 were detected as risk factors (Fig 4A). To evaluate the potential redundancy in gene expression profiles, pairwise Pearson correlations were computed for 14 genes and no gene pairs showed high correlation (r > 0.9), which indicated the absence of multicollinearity for these genes (Fig 4B).

Subsequently, in order to construct the prognostic model, the LASSO Cox regression was applied on 14 MCC genes from univariate Cox regression, and eight genes were identified with nonzero coefficients (S2 Fig). A risk score was calculated for each patient, and median and optimal cutoff were identified. Patients were stratified into high- and low-risk groups based on the median value of the risk score or an optimal cutoff threshold. Due to the close value of median and optimal cutoff (Fig 4C), and the widespread use of median value, median value was used for further analysis.

Among the eight genes, branched chain amino acid transaminase 1 (BCAT1), double PHD fingers 1 (DPF1), cyclin dependent kinase inhibitor 2B (CDKN2B), cyclin dependent kinase inhibitor 2C (CDKN2C) and tubulin alpha 3c (TUBA3C) had high expression in high-risk group, while insulin like growth factor 1 (IGF1), cell division cycle 14B (CDC14B) and SWI/SNF related BAF chromatin remodeling complex subunit ATPase 2 (SMARCA2) had high expression in low-risk group (Fig 4D). Kaplan-Meier analysis showed that the high-risk group had significantly poorer overall survival compared to the low-risk group for both median value and optimal cutoff (Fig 4E and S3 Fig). The 1- year, 3-year and 5-year AUC values were 0.772 and 0.681 and 0.667, respectively (Fig 4F and S3 Fig).

To evaluate whether the risk score could serve as an independent prognostic factor, univariate and multivariate Cox regression analyses were performed with risk score and clinical parameters: AJCC stage, age, grade, and gender. In univariate Cox regression analysis, stage and the risk score were found to be significantly associated with overall survival (FDR < 0.001), while age, gender, or grade did not show any significant associations (Fig 4G). To further assess the independence of the risk score, a multivariate Cox regression was performed with stage and risk score, which showed the risk score as an independent predictor of poor overall survival (HR = 3.085, 95% CI: 2.034–4.679, FDR < 0.001). Schoenfeld residual testing was performed for univariate and multivariate Cox regressions, which confirmed that all covariates satisfied the PH assumption, except for the risk score (p ≤ 0.05), which indicated prognostic effect may change over time.

C-index for stage (stage alone), risk score (risk score alone), and their combination (risk score + stage) were calculated. the C-index was 0.614 for stage, 0.691 for the risk score, and 0.719 for their combination. These results suggested that the risk score added prognostic value into standard clinical staging.

### Validation of the prognostic MCC gene signature

**Fig 5:**
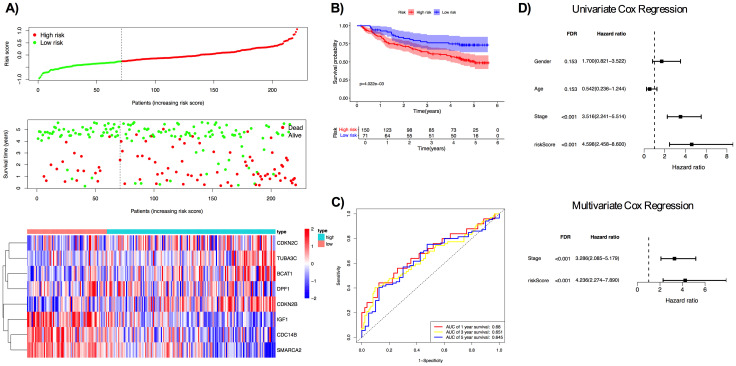
Evaluation of the prognostic performance of MCC gene signature in GSE14520 dataset. A) Distribution and survival information of the risk score and heat map of the gene expression of 8 genes. B) Kaplan-Meier analysis of HCC patients. C) ROC curve of HCC patients. D) Forest plots from univariate and multivariate Cox regression.

To validate the prognostic gene signature, GSE14520 dataset was used (Fig 5A). High-risk group showed poor overall survival in Kaplan-Meier analysis both for median value and optimum cutoff (Fig 5B and S3 Fig). The performance of MCC gene signature was evaluated by AUCs at 1-, 3- and 5- year, which were 0.68, 0.651 and 0.645, respectively (Fig 5C and S3 Fig). Similarly, risk score and stage emerged as independent prognostic factors based on univariate and multivariate Cox regression results (FDR < 0.001, Fig 5D). Unlike to TCGA LIHC, Schoenfeld residual testing confirmed that all covariates satisfied the PH assumption for GSE14520 dataset, including the risk score (p > 0.05).

For the GSE14520 dataset C-index for stage (stage alone), risk score (risk score alone), and their combination (risk score + stage) were also calculated. the C-index was 0.624 for stage, 0.633 for the risk score, and 0.703 for their combination, confirming contribution of the risk score to prognostic value of stage.

### Kaplan-Meier Analysis and distribution of risk score in different clinical parameters

**Fig 6:**
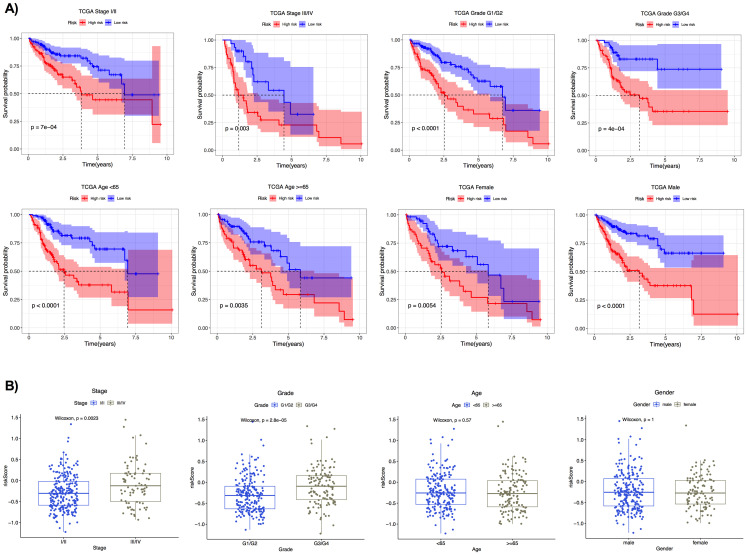
Kaplan-Meier analysis and distribution of the risk score in TCGA cohort with different clinical parameters. A) Kaplan-Meier plots for subgroups with different clinical parameters. B) Boxplots of the risk scores across the clinical subgroups.

The association between high-risk score and overall survival was evaluated in subgroups with different clinical parameters; AJCC stage, tumor grade, age, and gender in the TCGA cohort. In each subgroup, the high-risk group consistently showed significantly worse survival than the low-risk group (Fig 6A). These results confirm the prognostic value of the risk score in all subgroups. The higher risk score was observed in patients with more advanced stage (Stage III/IV) and grade (G3/G4) tumors (Fig 6B), suggesting biological relevance to HCC aggressiveness (Fig 6B).

## Discussion

In this study, to comprehend the various aspects of cell cycle and mainly focus on the MCC process, GOBP_MITOTIC_CELL_CYCLE gene set, covering the diverse features of MCC, such as cell division, cell cycle regulation and chromosome segregation, was selected and the differentially expressed MCC genes were identified. The diagnostic value of these genes in HCC was shown by two different ML methods, SVM-RFE and RF-RFE, and prognostic gene signature was generated by LASSO Cox regression.

ML algorithms facilitated the prediction of cancer progression, metastasis risk and response to treatment [37]. Several data types, including electronic health records, genomic and transcriptomic data, and medical images, can be effectively utilized for diverse purposes in cancer research, such as identifying diagnostic cancer biomarkers or co-deletion mutations, personalized medicine, and early detection. [37–40]. The utility of ML algorithms extends to structural biology, such as predicting protein crystallization from feature selection methods and ML algorithms [41], which highlights the broad adaptability of these approaches in health sciences.

ML methods, including LASSO, RF and SVM-RFE, have been used to predict tumor grades and discriminative features using different data sources, such as CT images and gene expression datasets [10,42]. Studies have applied SVM-RFE in combination with RF or other ML methods to gene expression data to identify diagnostic HCC biomarker candidates. Yi et al. combined SVM-RFE and LASSO on ferroptosis-related genes and achieved AUCs ranging from 0.879 to 0.785 for selected features in the training set, but did not report validation set for ML [43]. Zhou et al. used SVM-RFE with combination of other ML methods to identify important features from Notch signal-related genes, and reported AUC values as 0.908 ± 0.016 and 0.866 ± 0.064 for training and testing sets, respectively, for SVM classifier, while AdaBoost achieved the best diagnostic performance (AUC = 0.934) in the testing set [44]. Other studies used DEGs rather than specific biological processes or pathways to identify diagnostic biomarker candidates [10,45]. Gupta et al. used cell line models, instead of primary tumors, and obtained top 20 features using SVM and RF, RFE application resulted in three novel biomarkers with accuracy of 0.97. Filtration steps, removing transcripts with low expression and high correlation, were performed similar to current study. Among the different ML methods, SVM and RF had the best performance to find biomarkers for HCC [10]. Combination of SVM-RFE, RF, LASSO and WGCNA were employed to find diagnostic genes, the AUCs of each gene ranged between 0.961 and 0.877 in the validation set, although performance of the classifiers was not reported [46]. Similarly, there were studies focusing on individual features with over an AUC, such as 0.7 or 0.85, obtained from the intersection of SVM-RFE and LASSO, and did not evaluate classifier performance [45,47,48]. The current study distinguished from previous studies by stringent feature selection steps, including correlation filtering, RFE with 50 iterations to retain genes selected in ≥90% of runs, and a final RFE with 10-fold repeated cross-validation. The inclusion of internal and external validation sets improved the diagnostic accuracy. Permutation tests further confirmed model validity, showing observed AUCs were separated from the null distribution across all datasets. SVM-RFE model with the refined feature set from MCC gene list achieved high performance in TCGA (AUC = 1.0, mean AUC = 0.996, SD = 0.006), GSE77509 (AUC = 0.95) and in GSE144269 (AUC = 0.879) and the performance of the classifier with MCC genes was higher than similar studies in HCC with SVM-RFE to identify diagnostic genes by using gene expression data. The results of this study showed that SVM-RFE suppressed the performance of RF-RFE in the internal and external validation sets with AUC = 0.825 and AUC = 0.714, respectively.

The nine genes, TRIP13, RACGAP1, CDKN3, FBXO43, EZH2, SPDL1, TUBE1, CDC6 and E2F1, were common to SVM-RFE and RF-RFE and considered as diagnostic marker candidates for HCC. The high individual AUC values (AUCs > 0.81) of nine genes in TCGA, GSE77509 and GSE144269 also showed strong diagnostic power of these genes. While the downregulation of TUBE1 in HCC has been previously reported [49], current results with SVM-RFE and RF-RFE were the first to suggest the potential utility of TUBE1 as a diagnostic biomarker and highlight the role for TUBE1 in distinguishing HCC from normal tissue. Gene expression alterations of TRIP13, CDKN3, FBXO43, SPDL1 and E2F1 have been reported in HCC and these genes were proposed as prognostic markers or therapeutic targets for HCC patients [50–55]. The results of this study support the role of these genes as diagnostic biomarkers, expanding their clinical relevance. Among the nine candidate genes, RACGAP1, CDC6 and EZH2 have been proposed as diagnostic biomarkers for HCC [56–58], consistent the results of SVM-RFE and RF-RFE.

Previously prognostic gene signatures have been generated using LASSO Cox regression from cell cycle related genes for HCC [3,4,59]. In this study, 8-gene model was established by using LASSO Cox regression from genes particularly related to MCC process, due to its importance in tumorigenesis. In the TCGA cohort, the model achieved AUCs of 0.772, 0.681, and 0.667 at 1-, 3-, and 5-years, respectively. In the GSE14520 cohort, the AUCs were 0.68, 0.651, and 0.645, demonstrating robustness across datasets. The signature from Hallmarks of cancer gene sets from MSigDB to develop a cell cycle progression-derived model achieved AUCs of 0.776, 0.697, and 0.619 (for 1-, 3-, and 5-years, respectively) in TCGA, and 0.779, 0.803, and 0.762 in the LIRI-JP cohort [3]. Six cell cycle related MSigDB gene sets were used to establish a 13-gene prognostic model, which showed a high performance with AUC values of 0.835, 0.822, 0.808, 0.821, and 0.826 at 1-, 2-,3-, 4- and 5-years, respectively [4]. The 6-gene prognostic model from manually curated a literature-based list of 50 cell cycle genes achieved AUCs of 0.737, 0.712, and 0.683 (for 1-, 2-, and 3-years, respectively) in TCGA, and 0.742, 0.743, and 0.741 in the ICGC cohort [59]. Compared to these studies, current study demonstrated similar or slightly lower predictive accuracy, while emphasizing biological specificity to MCC process. It should be noted that time-dependent AUCs in the 0.6–0.7 range have been commonly observed for the validation sets for prognostic gene signatures [60,61]. Current study with prognostic MCC gene signature distinguished from most of the previous publications with similar set up due to its intense gene filtering at the univariate Cox regression, due to inclusion of PH assumption testing, avoidance of multicollinearity, consideration in median and optimal cutoff risk scores for patient stratification and evaluation of clinical relevance with respect to C-index and integration with AJCC stage.

Staging liver tumors, including AJCC TNM stage and histological grade, are accepted as an invaluable tool for predicting prognosis and therapy [62]. The model showed prognostic value independent of clinical parameters and its combination with AJCC stage improved prognostic discrimination, with C-indices increasing from 0.614 (stage) and 0.691 (risk score) to 0.719 (combined) in TCGA, and from 0.624 (stage) and 0.633 (risk score) to 0.703 (combined) in GSE14520. This improvement in C-index values indicates that the risk score added independent prognostic value to AJCC stage. Kaplan-Meier plots showed that the high-risk score was consistently associated with significantly poorer overall survival in all clinical subgroups, including early (Stage I/II) and advanced (Stage III/IV) stages, low (G1/G2) and high (G3/G4) tumor grades, younger and older age groups, and both sexes. These results indicate that prognostic gene signature might have broad applicability in clinical practice. Moreover, risk score were significantly higher in patients with advanced-stage and high-grade tumors, supporting a potential biological association with tumor aggressiveness.

This study provided diagnostic HCC biomarkers and a novel gene signature, which were established and validated by computational methods. Therefore, the genes identified in this study can be utilized to design novel in vivo studies that can evaluate the biological and mechanistic consequences of these gene expression alterations. Despite the strengths of this study, such as the integration of rigorous feature selection steps, and multi dataset validation, it is important to acknowledge the limitations. This study relied on retrospective datasets from public repositories. Although, it is common in computational studies, more datasets from prospective patient cohorts should be evaluated. In addition, no experimental validation was performed to confirm the identified diagnostic or prognostic genes. To experimentally validate the diagnostic and prognostic significance of the identified genes, future studies could examine their expression in HCC versus normal tissues using qPCR and immunohistochemistry. In addition, functional assays using siRNA-mediated knockdown of candidate genes in HCC cell lines may help to explain their roles in tumor proliferation and progression. It is also important to note that this study did not incorporate analysis of DNA driver mutations, which may provide additional insights into HCC biology and clinical outcomes. Therefore, future studies, which integrate genomic and transcriptomic datasets, could provide a more comprehensive understanding of diagnostic and prognostic mechanisms in HCC.

## Supporting information

S1 Fig Workflow of the study. (PDF)

S2 Fig 10-fold cross-validation for tuning parameter selection in the LASSO Cox regression and LASSO coefficient profiles. (PDF)

S3 Fig KM plots and ROC curves of TCGA LIHC and GSE14520 patients for optimal cutoff threshold. (PDF)
