# Supplementary figures and images for: Machine learning-based identification of diagnostic and prognostic mitotic cell cycle genes in hepatocellular carcinoma
Source: PLoS One. 2025 Aug 28;20(8):e0331118. doi: 10.1371/journal.pone.0331118 (PMC12393733; doi:10.1371/journal.pone.0331118)

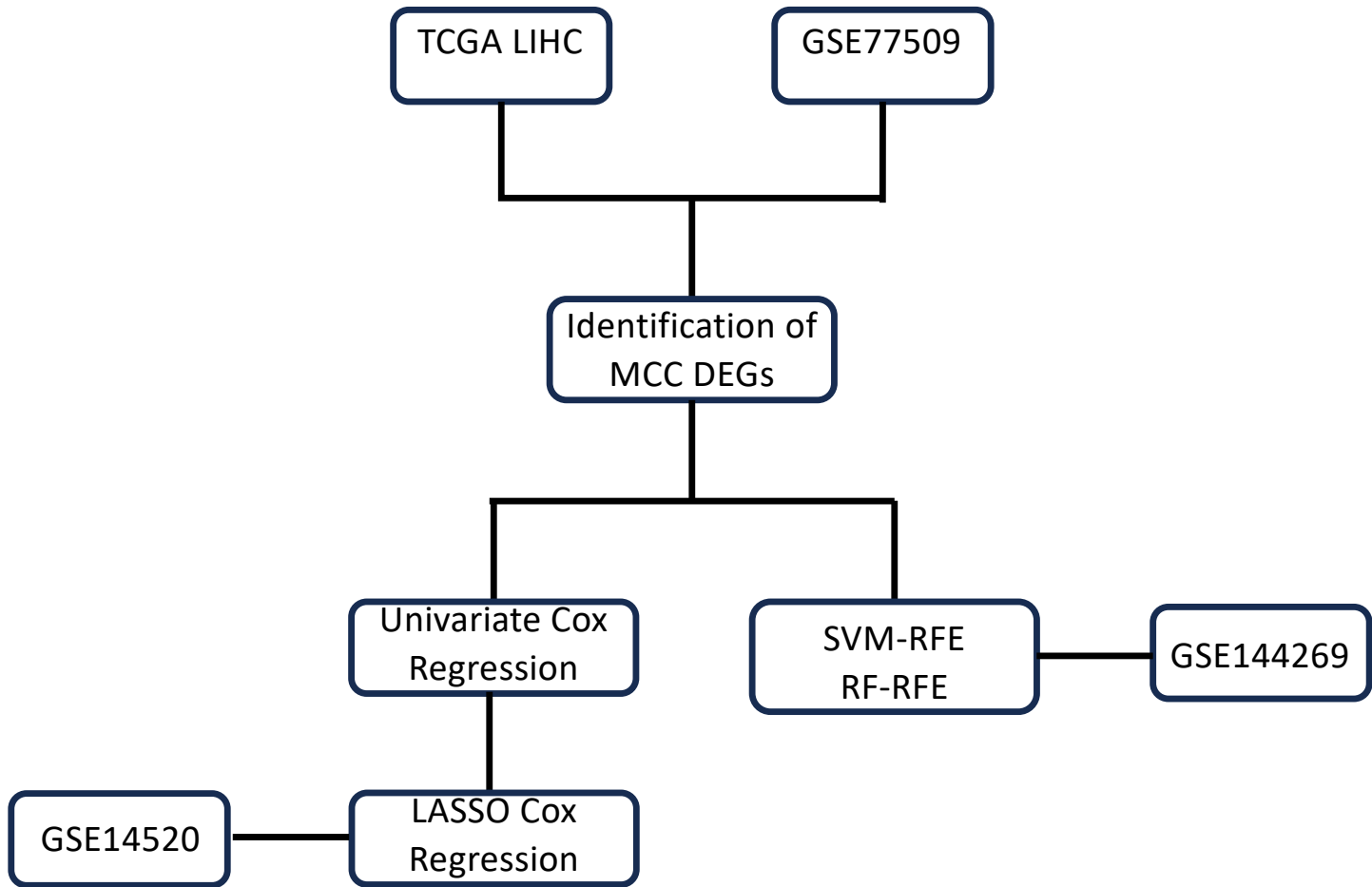

Supplement: S1 Fig — (PDF) [file pone.0331118.s001.pdf]

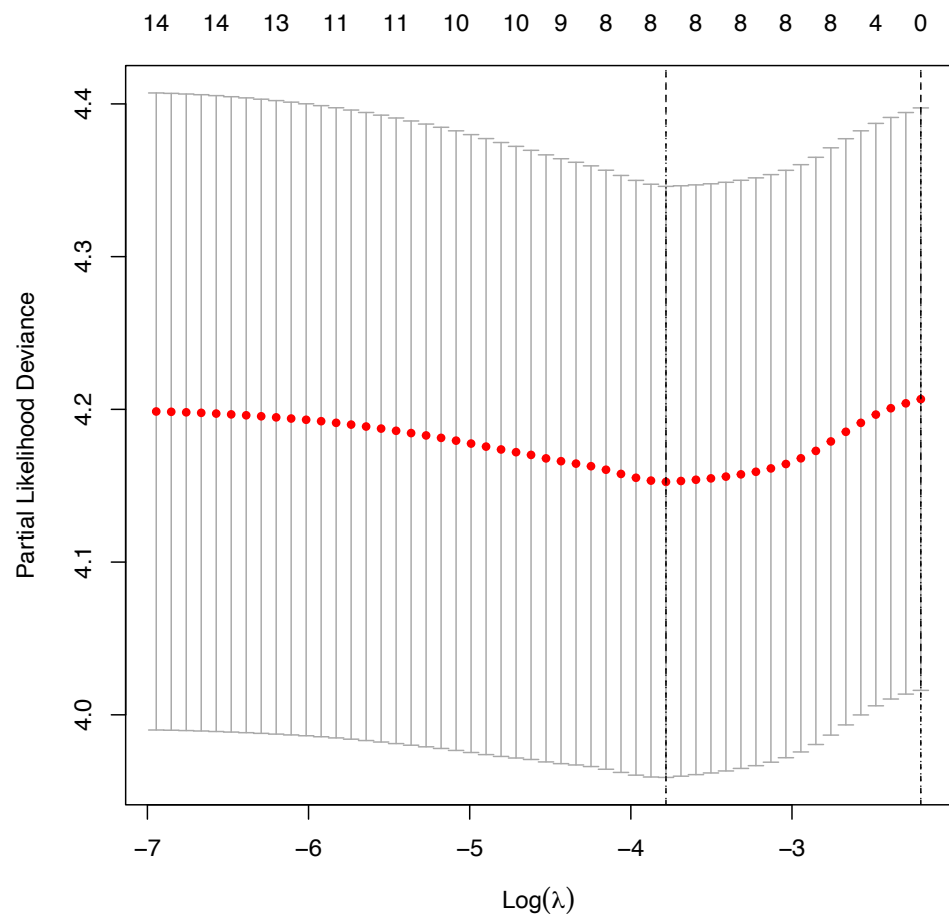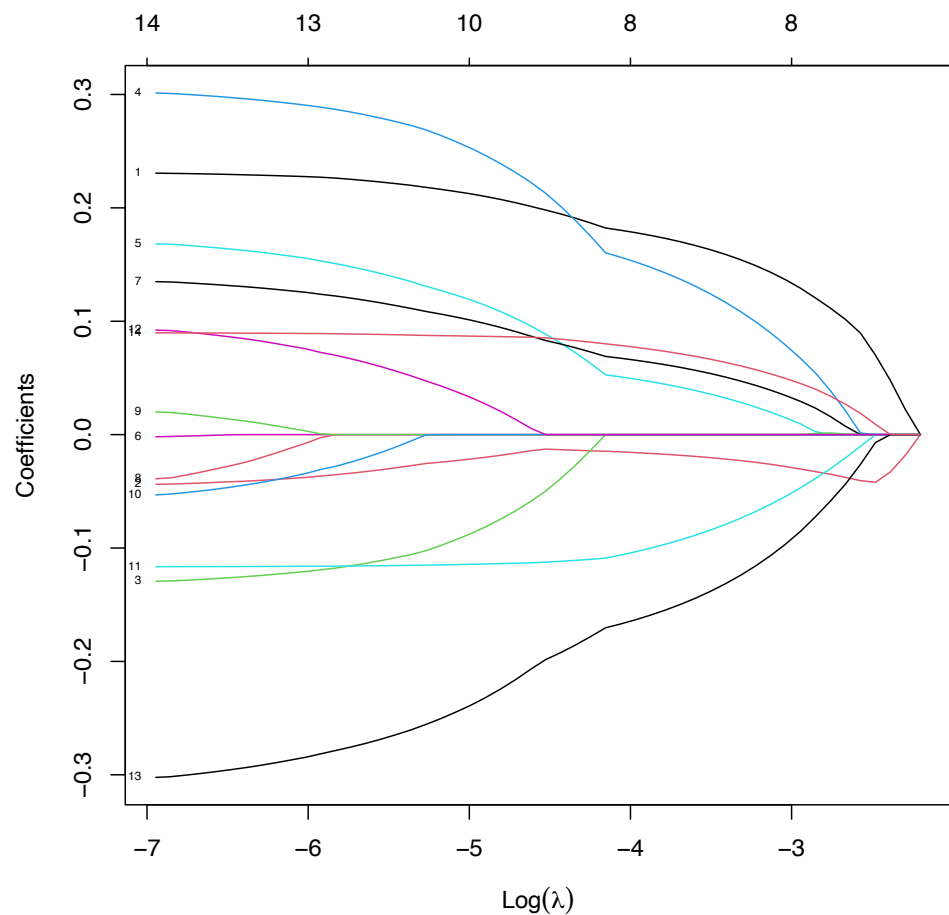

Supplement: S2 Fig — (PDF) [file pone.0331118.s002.pdf]

## TCGA

Risk High Low

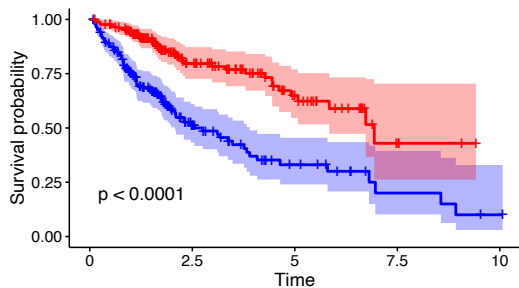

|      |      |     |     |    |     |    |
|------|------|-----|-----|----|-----|----|
| Risk | High | 173 | 39  | 14 | 4   | 1  |
|      | Low  | 169 | 68  | 26 | 4   | 0  |
|      |      | 0   | 2.5 | 5  | 7.5 | 10 |

## GSE14520

Risk High Low

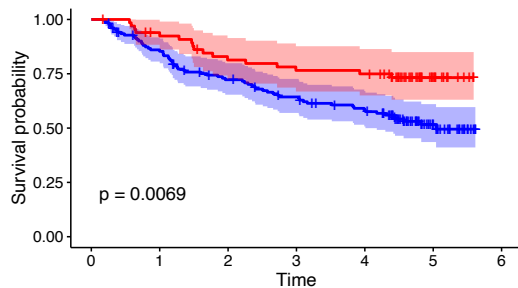

|      |      |     |     |     |    |    |    |   |
|------|------|-----|-----|-----|----|----|----|---|
| Risk | High | 154 | 127 | 102 | 88 | 76 | 26 | 0 |
|      | Low  | 67  | 60  | 51  | 48 | 47 | 15 | 0 |
|      |      | 0   | 1   | 2   | 3  | 4  | 5  | 6 |

## TCGA

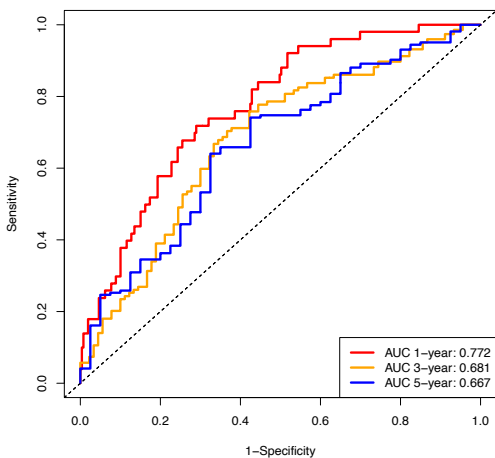

## GSE14520

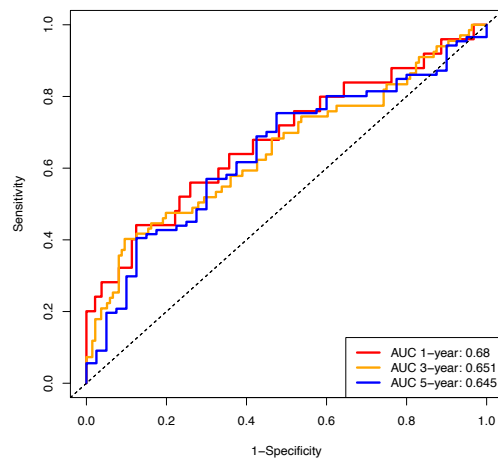

Supplement: S3 Fig — (PDF) [file pone.0331118.s003.pdf]
